# Impact of Sleep and Associated Factors on the Prevalence of Early Childhood Caries: An Analytical Cross-Sectional Study

**DOI:** 10.7759/cureus.81777

**Published:** 2025-04-05

**Authors:** Chhaya Patel, Megha C Patel, Swasti S Joshi, Miral Mehta, Disha Makwani, Foram Patel, Jessica Bale, Charmi Solanki

**Affiliations:** 1 Department of Pedodontics and Preventive Dentistry, Karnavati School of Dentistry, Karnavati University, Gandhinagar, IND

**Keywords:** circadian rhythms, early childhood caries, oral hygiene, prevalence, sleep-wake patterns

## Abstract

Background: Early childhood caries (ECC) occur soon after the eruption of teeth and can progress rapidly, causing severe impact on the physical, physiological, and psychological well-being of the child. Irregularities in sleep patterns can result in decreased salivary flow rate and the immune response, which ultimately can affect the rate of progression of early childhood caries.

Aim: To assess the prevalence of early childhood caries and its association with sleep practices, the habit of frequent snacking, and the usage of phones and smart devices by children before bedtime in the age group of three to five years.

Methodology: An analytical cross‑sectional study was conducted among 349 preschoolers aged three to five years studying in preschools of Gandhinagar city, Gujarat, to assess the caries prevalence and associated factors such as sleep practices and oral hygiene practices of children, as well as their snacking practice. A validated, structured questionnaire was administered to parents/guardians, and then caries status was evaluated using the deft index for each participant. Data was systematically compiled and analyzed using IBM SPSS Statistics for Windows, Version 23 (Released 2015; IBM Corp., Armonk, New York, United States). The association between early childhood caries and sleep disorder, the habit of frequent snacking, the bedtime of the child, the screen time of the child before going to bed, and oral hygiene was tested by the chi-square independent test of association.

Result: The caries were prevalent among 238 (68.2%) children in the study population. Out of 238 children, 88 (37%) were found to have severe early childhood caries. The prevalence of caries was significantly associated with late bedtime (after 11), i.e., among 110 children (76.9%) (P=0.034), decreased sleep duration (p=0.027), extracurricular activity (p=<0.001), and late dinner time (P=0.034). Multivariate regression shows bedtime (B=1.024, p=0.003) and frequent snacking (B=-1.364, p=0.011) significantly impact deft scores (R²=0.042, p=0.001), with frequent snacking (OR=0.412) leading to elevated frequency of caries.

Conclusion: The present study demonstrates the intricate relationship between sleep practices, oral hygiene, and dietary habits with the prevalence of ECC. Irregular sleep patterns, frequent snacking, and excessive screen exposure before bedtime were identified as potential risk factors contributing to the increased burden of early childhood caries (ECC).

## Introduction

Early childhood caries (ECC) is considered a multifactorial condition that can be influenced by behavioral, biological, and socio-economic factors [1]. Globally, the prevalence of ECC is 39% to 62% [2]. ECC is prevalent in approximately 49.6% of the population in India [3]. Lack of proper oral hygiene, increased meal frequency of sugary foods, along with the presence of *Streptococcus mutans* in the oral cavity and less parental commitment to helping their child brush their teeth are predisposing factors for dental caries in children [4].

Sleep is a vital physiological process essential for the cognitive, behavioral, and physical development of children. A healthy sleeping habit boosts immunity and minimizes cardiac diseases, hormonal imbalances, and metabolic diseases. Nevertheless, disrupted sleep habits in children have been associated with many negative health outcomes, particularly by highlighting their potential role in cardiac metabolic factors [5]. Potential paths by which sleep can affect the risk of caries include an abnormal flow of saliva caused by altered circadian rhythm, decreased IgA secretion, elevated salivary amylase activity, increased salivary interleukin-6, and higher *Streptococcus mutans* counts [6].

Most often, increased screen time in children leads to a decline in sleep duration, which can adversely affect the body's physiological balance, cognitive function, and overall health outcomes [7]. Moreover, an unhealthy lifestyle characterized by excessive screen time may negatively influence oral health behaviors, including frequency of tooth brushing, adherence to regular dental visits, and overall oral hygiene practices, which ultimately leads to poor oral health [8].

Also, recent shifts in dietary patterns across all age groups have shown a significant increase in the consumption of energy-dense, nutrient-poor snacks, contributing to over 30% of the daily energy intake in children [9]. Frequent consumption of such diets may lead to a recurrent decline in pH from carbohydrate fermentation over the tooth surface by the oral microbiome, causing dental caries [10]. The World Health Organization has identified dietary guidance as a key challenge in reducing dental caries along with diseases like coronary artery disease [11].

The American Academy of Pediatric Dentistry (AAPD) recommends tooth brushing twice a day to prevent caries. Tooth brushing on a daily basis is the most effective way of mitigating dental caries and gingivitis [12]. Inadequate oral hygiene practices combined with alterations in dietary habits can disrupt the normal microbiota of the oral cavity, which results in the pathogenesis of dental caries [13].

Several studies have analyzed the relationship between ECC, sleep patterns, and lifestyle behaviors [14-16]. However, evidence is available on the combined impact of sleep practices, screen time, extracurricular activities, the habit of frequent snacking, and nighttime oral hygiene practices on ECC. The current cross-sectional study aimed to bridge this gap by exploring the multifactorial relationship between these behavioral factors and ECC, with special emphasis on the role of sleep patterns in children's oral health.

## Materials and methods

Study design

A cross-sectional study was conducted at the Karnavati School of Dentistry, Gandhinagar, Gujarat, for which ethical approval was taken from the committee with ethical number KSDEC/23-24/Apr/013. The observational study was planned for four months to evaluate the prevalence of ECC and its relation with sleep-wake patterns and other factors in children aged three to five years.

Sample size

The sample size was estimated by the IBM SPSS Statistics for Windows, Version 23 (Released 2015; IBM Corp., Armonk, New York, United States) based on a study by Geethapriya et al. at a significance level of 5% and a power of test (Z) of 80% [1]. The formula for sample calculation in the current study was \[ \frac{Z^2*p*(1-p)}{e^2}\]

where the significance level was set at α=0.05 at the power (Z) of 95%, prevalence (p) was 0.5, and the margin of error (e) was set at 0.0586, which resulted in an estimated sample size of 349.

Participants

This was a multicenter cross-sectional study in which children in the age group three to five years with and without early childhood caries were included in our study. Participation in the study was based on voluntary consent, with all information collected remaining anonymous and confidential. Children with any systemic condition and those who were not willing to participate were excluded from the study. 

Sample selection and randomization

Preschools and schools in Gandhinagar city, Gujarat, were selected using a convenience sampling method, considering accessibility, willingness to participate, and administrative approval. Preschools that granted permission and had a sufficient number of children in the target age group (three to five years) were included in the study. Two preschools and three schools were selected, and the children meeting the inclusion criteria were selected by the cluster sampling method, in which one whole class of preschoolers was selected. A total of 349 children were assessed based on inclusion and exclusion criteria.

Procedure

A validated closed-ended questionnaire (Appendix 1) was prepared in accordance with the study by Geethapriya et al., which was done to assess sleep patterns, bedtime, snacking patterns, screen time, and maintenance of the child's oral hygiene [1]. The final set of questions was produced after a group discussion with renowned and distinguished pediatric dentists and was made available in both English and the native language. The questionnaire (Appendix 1) also included demographic details of participants. The reliability of the questionnaire was assessed through internal consistency analysis using Cronbach’s alpha analysis. Prior to oral examination, parents/caregivers were informed of the study, after which consent was obtained in writing. The questionnaire (Appendix 1) was distributed, and participants were instructed to complete all 14 questions, ensuring comprehensive responses.

Following that, teeth were cleaned and dried with sterile cotton before oral examination. The principal investigator evaluated the caries status using a mouth mirror and probe under natural sunlight and documented it using the deft and defs index. The deft index represents the count of decayed, extracted, and filled primary teeth, whereas defs indicates decayed, extracted, and filled surfaces, which were recorded for each child in the study [17]. The probable highest score for the deft index is 20, and the defs index is 88. Children with a deft score of one or more than one were categorized into the ECC group, while those with a deft score of zero were classified into the non-ECC group. The severity of ECC was classified based on AAPD criteria, with S-ECC defined by age-specific defs thresholds (≥4 at 36-47 months, ≥5 at 48-59 months, and ≥6 at 60-71 months) or the presence of smooth surface caries in maxillary anterior teeth [17].

Statistical analysis

All the data was gathered after properly assessing the answers to the questionnaire. The data collected was analyzed by the IBM SPSS Statistics for Windows, Version 23 (Released 2015; IBM Corp., Armonk, New York, United States). Based on the assessment of data distribution through Q-Q plots, histograms, and box plots, the dataset exhibited a left-skewed graph. Consequently, a statistical analysis was conducted using a non-parametric chi-square test to assess the association of sleep patterns and other factors in children with and without ECC. The continuous variables such as deft and defs scores were analyzed and categorized on the basis of AAPD criteria, which suggest any sign of decay in a child under three years old or, for children aged three to five, one or more cavitated, missing (due to caries), or filled smooth surfaces in primary maxillary anterior teeth, or a decayed, missing, or filled score of ≥4 (age three), ≥5 (age 4), or ≥6 (age 5) surfaces [17]. To ascertain the role of each component in developing early childhood caries, multivariate regression analysis was done. Statistical significance was defined at p< 0.05. To evaluate the risk of screen time and the habit of frequent snacking associated with early childhood caries, the odds ratio was determined.

## Results

Characteristic of the study population

A total of two preschools and three schools were visited, with 349 children participating in the study, with 291 (83.4%) aged five years. The total sample comprises 172 (49.3%) males and 177 (50.7%) females (Table 1). The predominant bedtime among participants was after 11 PM, i.e., 166 (47.6%) children, 9-11 PM, i.e., 137 children (39.3%), and 13 children (3.7%) with irregular bedtimes. Around 243 (69.6%) participants reported a nighttime sleep duration of six to eight hours, whereas 99 (28.4%) participants slept for more than eight hours. The majority of children were exposed to screens before sleep, with 193 (55.3%) participants having screen time of up to one hour and 37 (10.6%) participants exceeding two hours before bedtime (Table 1). Unhealthy dietary habits were found in 249 (71.3%) of participants, with 143 (41.0%) having dinner late, after 9 PM. Concerningly, 329 (94.3%) children skipped oral hygiene practices before bedtime.

**Table 1:** Characteristics of the study population n: total number of participants (n=349)

Characteristic	n=349	%
Age		
3 years	21	6.0%
4 years	37	10.6%
5 years	291	83.4%
Gender		
Male	172	49.3%
Female	177	50.7%
Extracurricular Activity		
Yes	80	22.9%
No	269	77.1%
Bedtime		
9	33	9.5%
9-11	137	39.3%
After 11	166	47.6%
Irregular	13	3.7%
Total Sleep Duration (1-2 years)		
<9 hours	15	4.3%
9-11 hours	221	63.3%
>11 hours	113	32.4%
Current Nighttime Sleep Duration		
<6 hours	7	2.0%
6-8 hours	243	69.6%
>8 hours	99	28.4%
Current Daytime Sleep Duration		
<2 hours	176	50.4%
2-3 hours	154	44.1%
>3 hours	19	5.5%
Specific Sleep Schedule Maintained		
Yes	61	17.5%
No	288	82.5%
Screen Time Before Bed		
≤1 hour	193	55.3%
2 hours	106	30.4%
>2 hours	37	10.6%
No screen time	13	3.7%
Use of Mobile/Smart Devices Before Bedtime		
Yes	297	85.1%
No	52	14.9%
Dinner Time		
7-8 PM	37	10.6%
8-9 PM	169	48.4%
After 9 PM	143	41.0%
Habit of Frequent Snacking		
Yes	249	71.3%
No	100	28.7%
Nighttime Oral Hygiene Practices (Brushing)		
Yes	20	5.7%
No	329	94.3%

Prevalence and severity of early childhood caries

The caries status of the study participants was assessed based on the deft index (decayed, missing, and filled primary teeth), following the WHO criteria. Out of the total study population, 238 (68.2%) children exhibited ECC (deft>0), while 111 (31.8%) participants were caries-free (deft=0) (Table 2).

**Table 2:** Prevalence of early childhood caries (ECC) n: total number of participants (n=349)

Characteristic	n	%
ECC Status		
Present (deft>0)	238	68.2%
Absent (deft=0)	111	31.8%
Mean deft (±SD)=3.8 (±4.2)		

Caries prevalence and severity increase with age. Out of 238 children examined (n=238), 88 children were found to have severe early childhood caries (S-ECC) (Table 3) based on the American Academy of Pediatric Dentistry (AAPD) criteria. The 60-71 months (five years) group shows 107 cases of ECC, whereas 56 S-ECC cases, accounting for 68.5% of total cases (Table 3). In contrast, the 36-47 months (three years) group has fewer cases but 15 cases of S-ECC, which are more than ECC, indicating early onset of severe caries (Table 3).

**Table 3:** Distribution of ECC and S-ECC n: total participants diagnosed with early childhood caries (n=238); ECC: early childhood caries; S-ECC: severe early childhood caries

Age	Severity (n=238)		%
	ECC	S-ECC	
36-47 months (3 years)	10	15	10.5%
48-59 months(4 years)	33	17	21.0%
60-71 months (5 years)	107	56	68.5%

Association of early childhood caries with sleep patterns and other risk factors

ECC was significantly associated with later bedtimes (p=0.034), sleep durations at night (p=0.027), longer screen times (p=0.038), and usage of mobile phones before bed (p=0.041) (Table 4). Late dinners, particularly after 9 PM (p=0.034), frequent snacking (p<0.001), and extracurricular activity (p<0.001) were significantly associated with caries, whereas nighttime oral hygiene practices had no significant impact (Table 4).

**Table 4:** Non-parametric chi-square test for association of sleep patterns, diet practices, oral hygiene practices, and other factors with ECC status Statistically significant at p<0.05; χ²: chi-square test; ECC: early childhood caries

Variable	ECC Present (n=238)	ECC Absent (n=111)	χ² value	p-value
Bedtime			6.742	0.034
9 PM	20 (54.1%)	17 (45.9%)		
9-11 PM	108 (63.9%)	61 (36.1%)		
After 11 PM	110 (76.9%)	33 (23.1%)		
irregular	7 (53.8%)	6 (46.2%)		
Total Sleep Duration (1-2 years)			1.893	0.388
<9 hours	12 (80.0%)	3 (20.0%)		
9-11 hours	148 (67.0%)	73 (33.0%)		
>11 hours	78 (69.0%)	35 (31.0%)		
Current Nighttime Sleep Duration			7.216	0.027
<6 hours	7 (100%)	0 (0%)		
6-8 hours	171 (70.4%)	72 (29.6%)		
>8 hours	60 (60.6%)	39 (39.4%)		
Current Daytime Sleep Duration			2.105	0.349
<2 hours	115 (65.3%)	61 (34.7%)		
2-3 hours	109 (70.8%)	45 (29.2%)		
>3 hours	14 (73.7%)	5 (26.3%)		
Specific Sleep Schedule Maintained			0.012	0.914
Yes	41 (67.2%)	20 (32.8%)		
No	197 (68.4%)	91 (31.6%)		
Screen Time Before Bed			8.453	0.038
≤1 hour	122 (63.2%)	71 (36.8%)		
2 hours	78 (73.6%)	28 (26.4%)		
>2 hours	31 (83.8%)	6 (16.2%)		
No screen time	7 (53.8%)	6 (46.2%)		
Use of Mobile/Smart Devices Before Bedtime			4.172	0.041
Yes	209 (70.4%)	88 (29.6%)		
No	29 (55.8%)	23 (44.2%)		
Dinner Time			6.742	0.034
7-8 PM	20 (54.1%)	17 (45.9%)		
8-9 PM	108 (63.9%)	61 (36.1%)		
After 9 PM	110 (76.9%)	33 (23.1%)		
Habit of Frequent Snacking			12.576	<0.001
Yes	184 (73.9%)	65 (26.1%)		
No	54 (54.0%)	46 (46.0%)		
Nighttime Oral Hygiene Practices (Brushing)			0.103	0.748
Yes	13 (65.0%)	7 (35.0%)		
No	225 (68.4%)	104 (31.6%)		
Extracurricular Activity			43.467	<0.001
Yes	34 (42.5%)	46 (57.5%)		
No	28 (10.4%)	241 (89.6%)		

Multivariate regression analysis reported that bedtime (B=1.024, p=0.003) and frequent snacking (B=-1.364, p=0.011) were significantly associated with increased deft scores (R²=0.042, p=0.001) (Table 5). The analysis explained 4.2% variance in deft (decayed, extracted, filled teeth) scores, which suggests a significant relation between bedtime and early childhood caries (Table 5).

**Table 5:** Multivariate regression analysis to predict the deft score The same was done from various parameters like the bedtime (PM) of the child at night, the habit of frequent snacking, the usage of mobile phones or any other smart devices before going to or during bedtime, and the total screen time of the child before going to bed. B: unstandardized coefficient; t: t statistic value; p-value: significance value - <0.05

Variable	Unstandardized Coefficients		Standardized Coefficients	t	p-value	95% Confidence Interval	
	B	Std. Error	Beta			Lower Bound	Upper Bound
(Constant)	1.234	1.480	-	0.834	0.405	-1.676	4.144
Bedtime (PM) of the child at night	1.024	0.337	0.163	3.039	0.003	0.361	1.687
Habit of frequent snacking	-1.364	0.533	-0.140	-2.559	0.011	-2.411	-0.316
Usage of mobile phones or any other smart devices before going to bed or during bedtime	0.894	0.607	0.080	1.472	0.142	-0.301	2.088
Total screen time of the child before going to bed	0.427	0.504	0.045	0.846	0.398	-0.565	1.418

The presence of a habit of frequent snacking (odds ratio=0.412), usage of mobile phones or any other smart devices before going to or during bedtime (odds ratio=0.708), and total screen time of a child before going to bed (odds ratio=0.663) are having more chances of occurrence of dental caries (Table 6).

**Table 6:** Odds ratio for habit of frequent snacking and screen time associated with early childhood caries

Parameters	Odds Ratio	95% Confidence Interval	
		Lower Bound	Upper Bound
Habit of frequent snacking	0.412	0.248	1.684
Usage of mobile phones or any other smart devices before going to bed or during bedtime	0.708	0.395	1.268
Total screen time of the child before going to bed	0.663	0.380	1.154

## Discussion

There is growing evidence that late bedtimes and irregular sleep patterns are related to an increased prevalence of early childhood caries (ECC), likely due to higher nighttime snack consumption and reduced salivary flow, which create a favorable environment for cariogenic bacteria. Throughout the first few years of life, sleep patterns undergo significant changes as overall sleep time declines with age, daytime sleep gradually fades, and sleep during the night persists and strengthens [18]. In the current study, children who slept late (after 9 PM), had a sleep duration of less than 11 hours, and had irregular sleep patterns were more prone to early childhood caries. Multivariate regression analysis (Table 5) demonstrated that children aged three to five years had a significant relationship with the prevalence of caries. The hormones that govern appetite are stimulated by delayed bedtimes, leading to higher food consumption. Excessive diet intake, along with decreased saliva at night, may cause increased accumulation of dental plaque and ultimately result in demineralization, causing decay in the tooth [19]. Zhou et al. reported that caries prevalence was lower in children in the age group three to five years who had longer sleep durations [20]. Using the Feeding at Sleep Time Scale, Ganesh et al. examined the relationship between ECC and sleep time feeding habits and found that, in children aged 12 to 36 months, the frequency of ECC increased with sleep time feeding patterns [21].

Global modernization has heightened sedentary behavior, especially in children and adolescents. Children and teenagers should not spend more than two hours a day in front of gadgets and screens, according to the American Academy of Pediatrics [22]. Our study observed that children with screen time exceeding one hour exhibited a greater susceptibility to tooth decay. These results align with the observations of Garg et al., who observed that prolonged screen exposure exceeding two hours was significantly associated with unhealthy dietary patterns, including the frequent consumption of sugar-rich and acidogenic foods, thus leading to a higher risk of dental caries development in primary dentition [8]. Silva et al. also linked extended screen time to higher intake of sugary and unhealthy snacks [23]. In our study, children with more than one hour of screen time were 0.663 times more likely to develop early childhood caries.

As a child's oral microbiome evolves with the eruption of new teeth, simple carbohydrates (e.g., lactose, sucrose, glucose) serve as essential substrates for cariogenic bacteria. Prolonged contact of these sugars with teeth increases the prevalence and incidence of dental caries [24]. The pathogenesis of ECC is intricately linked to the metabolic processes of dental biofilm bacteria, wherein the fermentation of dietary carbohydrates precipitates a marked decline in oral pH, fostering an acidic milieu conducive to enamel demineralization. This deleterious cascade is further potentiated by recurrent exposure to fermentable sugars, compounded by inadequate plaque control, thereby significantly elevating the risk of ECC manifestation [10]. A dose-response relationship exists between sugar intake and caries risk, with a steep increase in caries prevalence between 15 and 35 kg/person/year of sugar consumption [9]. Stephan and Miller (1943) noted that pH drops within 30 minutes of sugar intake, emphasizing the importance of timing in dietary habits [25]. The WHO also recommends decreasing the sugar intake to no more than 10-20% of total calories [26].

Inadequate oral hygiene, such as not brushing at night, can significantly impact a child's oral health [27]. In the current study, 94.3% of children did not brush at night. However, inadequate brushing was not found to be significantly related to the prevalence of caries in preschoolers. The AAPD advises brushing as soon as the first tooth erupts, with Retnakumari and Cyriac recommending that delayed brushing initiation (after 24 months) leads to more severe decay [28]. Delayed brushing creates an environment conducive to bacterial growth and caries. Parents must be educated on the importance of early and consistent tooth brushing.

Sleep and extracurricular activity are two of the most significant adaptable behaviors that help children move toward a healthy lifestyle [29]. In the current study, children involved in extracurricular activity had a decreased occurrence of early childhood caries as compared to other children. Children engaged in extracurricular activities demonstrated a more consistent sleep pattern, which is associated with a reduced prevalence of ECC, likely due to better-regulated circadian rhythm and adherence to a structured daily routine.

The cross-sectional design of the current investigation made it difficult to determine a causal association between risk factors and ECC. The use of questionnaire-based data collection may have introduced recall bias and underreporting, particularly in screen time, dietary habits, and oral hygiene practices. Using convenience sampling from selected preschools and schools may lead to selection bias, which can restrict the generalizability of the results. To confirm these results and establish threshold values for preventative interventions, larger sample sizes are needed in future longitudinal investigations. 

## Conclusions

Late bedtimes, inadequate sleep duration, poor oral hygiene, frequent snacking, and increased screen time at night have been identified as key factors that contribute to the progression of caries in children, in addition to other risk factors. These behaviors disrupt healthy routines and create an environment conducive to the growth of cariogenic bacteria.

A comprehensive approach is necessary to effectively address these issues, with a strong emphasis on parental counseling. It is crucial to educate parents on the significance of maintaining consistent oral hygiene practices twice a day, bedtime routines, and limiting screen time before sleep.

## Appendices

Appendix 1

Questionnaire

1.     Name :

2.     Age/sex:

3.     Number of family members

a.     Three

b.     >3

4.     Habit of frequent snacking

a.     Yes 

b.     No 

5.     Meal (dinner) time of child

a.     7-8

b.     8-9

c.     After 9

6.     Any oral hygiene practices performed at night ?

a.     Yes

b.     No 

7.     Usage of mobile phones or any other smart devices before going to or during bedtime

a.     Yes

b.     No

8.     Total screen time of child before going to bed

a.     1hrs

b.     2hrs

c.     >2hrs

9.     Specific sleep schedule maintained

a.     Yes

b.     No

10.   Bedtime of the child at night

a.     9

b.    9-11

c.    After 11

d.    Irregular

11.   Total duration of sleep between the ages 1- 2 years

a.    <9hrs

b.    9-11hrs

c.    >11hrs

12.  Daytime sleep duration of the child

a.    <2 hrs

b.    2-3hrs

c.    >3hrs

13.   Night time sleep duration

a.     <6 hrs

b.    6-8 hrs

c.    >8 hrs

14.   DEFT indices

  55           54          53           52          51          61          62          63           64          65

  85            84         83           82          81          71          72          73           74          75

Total score :-
